# Analyzing the Vibration Response of Adhesively Bonded Composite Cantilevers

**DOI:** 10.3390/ma18010093

**Published:** 2024-12-29

**Authors:** Jarosław Chełmecki, Paweł Szeptyński, Dorota Jasińska, Arkadiusz Kwiecień

**Affiliations:** 1 Deformations and Vibrations of Structures Laboratory, Cracow University of Technology, 31-155 Kraków, Poland; jaroslaw.chelmecki@pk.edu.pl; 2 Division of Structural Mechanics and Material Mechanics, Faculty of Civil Engineering, Cracow University of Technology, 31-155 Kraków, Poland; dorota.jasinska@pk.edu.pl (D.J.); arkadiusz.kwiecien@pk.edu.pl (A.K.)

**Keywords:** timber, composites, vibration, damping, adhesive bonding

## Abstract

In this study, we investigated the vibration of adhesively bonded composite cantilevers consisting of two beech wood lamella and a bondline of flexible polyurethane. The beams had a constant total height, while the thickness of the adhesive layer varied. We analyzed both the driven and free vibration of a single cantilever beam and a cantilever with an additional mass attached to its end. The eigenfrequencies were determined using Fourier analysis of a sweep load response, the response to an impact load excited using an impact hammer, and the response observed via the manual displacement of the beam’s tip. The system’s damping was estimated according to the recorded logarithmic decrement. Theoretical estimates of the fundamental natural frequency were obtained using the γ-method and employing a linear elastic theory of composite beams. A numerical modal analysis was carried out using the finite element method. Upon comparing the results of our experiments with the numerical estimates and theoretical predictions, a fair agreement was found.

## 1. Introduction

Timber structures are widely recognized as an economically efficient, environmentally friendly, and aesthetic alternative to the most common construction technologies, such as reinforced concrete (RC) casting, masonry, or erecting structures from steel or precast RC members. The constant development of the adhesive industry [1] and ongoing research into the mechanical performance, sustainability [2], and durability [3,4] of adhesive joints broaden the possible range of application of timber elements even further. In the design process, achieving sufficient stiffness in a timber structure, especially in the case of framework structures or systems utilizing adhesive joints, is frequently a decisive issue. This not only ensures that the requirements of the Serviceability Limit State are met but also guarantees the vibrational comfort of people inside the building. These problems have been examined in multiple studies carried out in recent decades; a review of these matters is presented in a recent study by Aloisio et al. [5].

Interestingly, the review mentioned above revealed practically no research devoted to adhesively bonded structures, except for a single study on hybrid adhesive-screwed connections when applied to timber–concrete composite (TCC) floors. The static deformation of adhesively jointed composite beams was the subject of several studies, including investigations of TCC beams [6,7,8,9,10], steel–concrete composite beams [11,12], glass–steel beams [13], and concrete–GFRP composites [14]. However, the dynamic response of adhesively bonded beams—to the authors’ best knowledge—requires investigation, and it seems that the vibration of adhesively bonded timber structures or structural elements is still not a well-recognized problem. This is the research gap investigated in this study.

While the vibration of adhesive lap joints has already been studied in multiple investigations (see, e.g., [15,16,17,18]), the number of scientific papers on the vibration of adhesively bonded composite beams is significantly smaller. García-Barruetabeña and Cortés [19,20] experimentally analyzed the influence of geometric parameters (overlap length and thickness of adhesive layer) on the dynamic response of metallic beams adhesively bonded to the supports, measuring the eigenfrequencies, amplitudes, and modal loss factor. Increasing the overlap length shortens the vibrating length of the beam while simultaneously increasing the system’s stiffness, contributing to an increase in eigenfrequencies and resonance peak amplitudes. A greater thickness of the joint results in less joint stiffness, leading to a reduction in eigenfrequencies. In [20], an FEM simulation of this system was carried out to numerically investigate the influence of the geometric parameters mentioned above and the mechanical properties of the adhesive. The previous conclusions were generally confirmed by FEA. The Young’s modulus of the adhesive was found to have a minor impact on the system’s eigenfrequencies. This research only partially addresses the problem of adhesively bonded beams vibrating, as it only covers cases of bonding the beams to the supports.

In [21], Rao and Crocker investigated the vibrations of graphite epoxy beams joined using an adhesive lap joint. The viscoelastic properties of the epoxy resin were incorporated into an analytical estimation of the system’s eigenfrequencies. The theoretical predictions exhibited excellent agreement with the experimental results for the three fundamental eigenfrequencies.

In [22], Adam, Ladurner, and Furtmüller developed a beam theory for composite elastic beams consisting of slender layers connected by an elastic joint, assuming that the interaction forces at the interfaces are proportional to the slip occurring at the interface. The theory accounted for moderately large deformations, as well as imperfections in the form of initial slight deflections. The time history predictions it provided were in very good agreement with the finite element analysis results. Similar investigations were carried out by Saito and Tani [23] and He and Oyadiji [24]. Saito and Tani provided a system for equations of motion and compared their obtained numerical predictions with two other approximate estimates, while He and Oyadiji investigated the influence that the mechanical properties of an adhesive have on the dynamic response of a cantilever consisting of two adhesively jointed plates. It was found that an increase in the adhesive’s Young’s modulus resulted in an increase in the system’s natural frequencies. The influence of the Poisson’s ratio was rather negligible. None of these articles, however, consider fully bonded composite beams.

Very few articles have focused solely on the dynamic response of adhesively bonded composite beams. Lasowicz and Jankowski investigated the possibilities of amplifying damping in a composite aluminum cantilever using flexible adhesive bonding [25]. Lasowicz, Kwiecień, and Jankowski [26] studied variations in the vibration response of an aluminum cantilever beam with a mass at its tip after applying a flexible polyurethane layer. They also examined the response of a composite beam composed of two aluminum bars bonded together with a layer of polyurethane adhesive. They found that the eigenfrequencies of the aluminum beam were not significantly affected by the addition of the adhesive layer, but the damping of the system increased by up to four times. Next, they analyzed the vibrations of composite beams when the thickness of the adhesive layer varied. The thickness of the adhesive had a minor effect on the value of the first eigenfrequency but had a somewhat greater influence on the value of the second, decreasing it as the layer thickness increased. A thicker adhesive layer also contributed to increased damping in the system for the first mode; however, the impact on the second mode was less clear.

The problem of cantilevers/columns with a tip mass vibrating has been addressed in multiple research papers that consider various analytical approaches [27,28,29], structural health monitoring [30], and the behavior of offshore structures [31]. None of these studies, however, investigated adhesively bonded structures.

This study contributes to the understanding of the vibration characteristics of adhesively bonded composite beams, which is still insufficient. Cantilevers (columns fixed in their bases) consisting of two beech wood lamella bonded with a layer of flexible polyurethane adhesive F&R^®^PS (producer FlexAndRobust Systems Sp. z o.o.: Cracow, Poland) were investigated. Four types of beams with differing adhesive layer thicknesses were analyzed, while the total height of the beam’s cross-section was kept constant. The vibrations were excited in a plane perpendicular to the plane of the adhesive layer. Three types of excitations were used: a sweep load applied through a shake table (for three levels of amplitude), an impact load applied with a modal hammer (in two variants: hitting the top of the cantilever and in the middle of its length), and the manual deflection of the beam tip. The tests were performed in two configurations: a sole composite cantilever and a cantilever with a lumped mass attached to its free end. The resonance frequencies were measured under sweep loads, along with the fundamental eigenfrequencies obtained from the free vibrations after impact loading. Additionally, the logarithmic damping decrement was determined from the free vibration response. The flexural stiffness of the adhesively bonded beams was investigated using a static four-point bending test; the obtained results were then applied to classical formulas for estimating the natural frequencies of cantilever beams. Theoretical and numerical estimates of fundamental natural frequencies were proposed, and a fair agreement between the theoretical predictions and experimental results was found.

## 2. Materials and Methods

### 2.1. Materials

**Figure 1:**
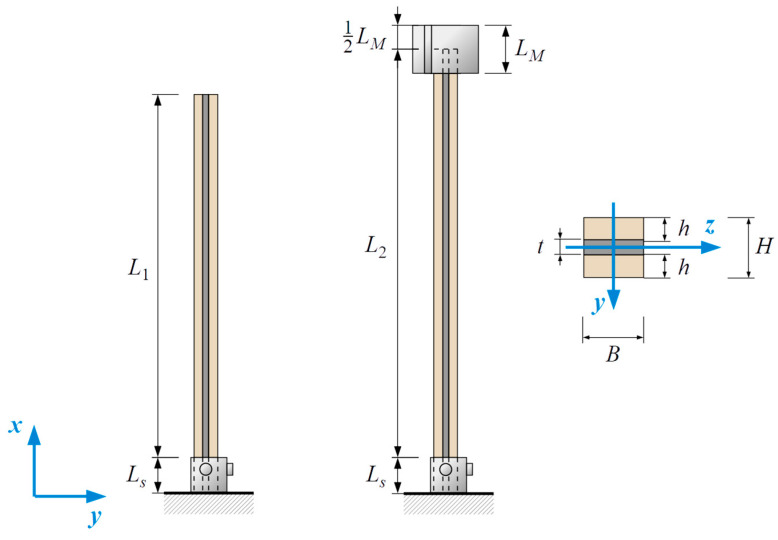
Geometry of the specimens.

The investigated beams were composed of two beech wood lamellas bonded with the use of a flexible F&R^®^PS polyurethane adhesive. The thickness of the adhesive layers varied between the different types of cantilevers (t=2, 10, 15, 20 mm), while the total beam height was H=50 mm in each case. The width of the specimen was B=50 mm. The total length of the specimens without additional mass was $L_{1}+L_{s}=1560\;mm$, where $L_{s}=50\;mm$ was used for fixing the supported end of the cantilever. Half of the tests were carried out with an additional mass of 5.93 kg attached to the free end of the cantilever. The height of the box composed of steel sheets was $L_{M}=100\;mm$ (see Figure 1). The total length of the specimens with the additional attached mass was $L_{2}+L_{s}=1650\;mm$.

The dimensions of each component part of the composite specimen cross-sections are given in Table 1.

Additionally, solid wood reference beams with a square cross-section of 50 mm×50 mm were also investigated in order to estimate the dynamic Young’s modulus of the wood.

#### 2.1.1. Beech Wood

The volumetric density of the beech wood was estimated as the mean value of the measurements performed for six specimens and was found to be $\rho_{w}=695\frac{kg}{m^{3}}$. The dynamic Young’s modulus was estimated by adjusting the mechanical parameters of a linear elastic orthotropic material in a numerical finite element model, reflecting the experimental results of a free vibration test carried out on the solid wood reference beams. The longitudinal stiffness modulus was found to be $E_{w,L}=12.91\;GPa$. The other components of the elasticity tensors were estimated by assuming that the ratios of these moduli to the longitudinal Young’s modulus were those provided in [32] (Table 2). For these values, the numerical first natural frequency of a timber beam $\left(\omega =15.214\;Hz\right)$ was in almost perfect agreement with the experimental value obtained as a response to excitation using an impact hammer $\left(\omega =15.213\;Hz\right)$.

Similarly, a good agreement was also found for the beams with an additional attached mass in terms of both the numerically predicted $\left(\omega =4.74\;Hz\right)$ and the measured natural frequencies $\left(\omega =4.93\;Hz\right)$.

The remaining Poisson ratios were determined according to the symmetry of the compliance tensor, as expressed in (1).

(1) $$\frac{\nu_{w,ij}}{E_{w,i}}=\frac{\nu_{w,ji}}{E_{w,j}},\;\;i,j=L,R,T$$

#### 2.1.2. F&R^®^PS Polyurethane Adhesive

The volumetric density of the polyurethane ($\rho_{a}=1419.9\;\frac{kg}{m^{3}}$) was determined using the weight of the composite beam with a 20 mm adhesive layer thickness. The Young’s modulus was assumed to be $E_{a}=27.97\;MPa$ based on the results of the uniaxial tension tests at the strain rate of 10 [1/min] published in [33]. The Poisson ratio for polyurethane was assumed to be $\nu_{a}=0.4$ [34,35].

### 2.2. Laboratory Setup

**Figure 2:**
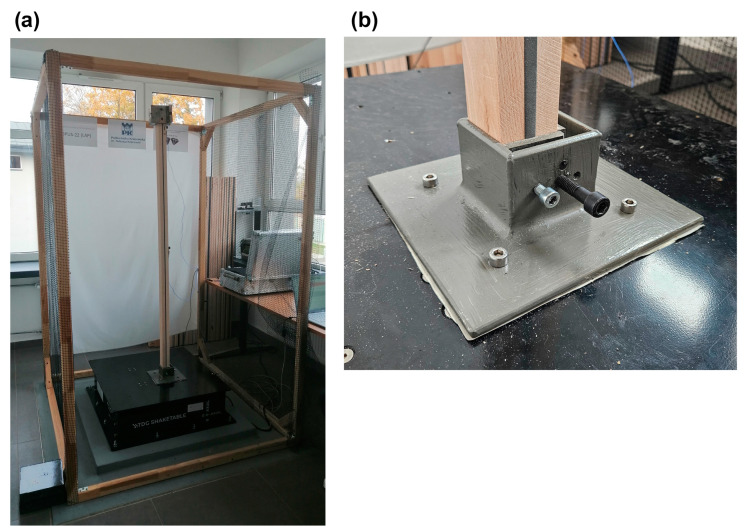
(**a**) Laboratory setup; (**b**) fixing of the specimen to the shake table.

All specimens were fixed to a QuakeLogic Bi-Axial-TDG Shake Table (QuakeLogic: Roseville, CA, USA); see Figure 2. Its range of frequencies was 0–20 Hz, and its range of applicable amplitudes was ±125 mm. The maximal values of amplitude depended on the frequency applied. The maximum admissible load at acceleration 1 g was 110 kg, and the maximal acceleration of excitation was 5 g. Two 356A16-type accelerometers were attached to each specimen, one in the middle and the other at the top of the specimen, which were provided by PCB Piezotronics (Depew, NY, USA). Their sensitivity was 10 mV/g, their measuring range was ±50 g (peak values) and the range of frequencies was 0.5–5000 Hz. During all performed measurements, the sampling frequency was set to 1024 Hz. Impact tests were carried out with an 086D20-type impact hammer manufactured by PCB Piezotronics. Its sensitivity was 0.23 mV/N, its measuring range was ±22240 N (peak value), its mass was 1.1 kg, and its resonance frequency was greater than 12 kHz.

### 2.3. Testing Procedures

A total of 56 tests were performed. The vibration response was recorded for two cases for each specimen—a sole beam and a beam with an additional mass attached to the top of the cantilever. Two types of excitations were used—the sweep load, using the shake table, and the impact load. Sweep loads were tested in two ranges of frequencies: 2–6 Hz for determining the beams with an attached mass and 10–17 Hz (for adhesive layers 2 mm and 10 mm thick) or 8–12 Hz (for adhesive layers 15 mm and 20 mm thick) for the sole cantilevers. Each sweep excitation was performed for three levels of acceleration amplitude. The reference amplitude was approximately 7 m/s*^2^*, and depending on the response of the specimen, the amplitude was altered in the next measurement. Each sweep lasted 120 s, with the frequency increasing linearly from the initial value up to the terminal value. Three types of impact load were employed: excitation with an impact hammer hitting the top of the cantilever, excitation with an impact hammer hitting the middle of the cantilever, and the manual deflection of the cantilever top.

The signals recorded by the accelerometers were filtered with a 7th-order Butterworth low-pass filter with a cutoff frequency of 30 Hz. The resonance frequencies in both the sweep and impact tests were determined as the peak values of the FFT spectrum of the accelerograms. The damping properties of the composite beams were estimated using the logarithmic damping decrement, which was determined by assessing the recorded signal starting from the first proper period to the period at which the amplitude was greater than 10% of the maximal amplitude.

### 2.4. Estimation of Flexural Stiffness in Four-Point Bending Test

**Figure 3:**
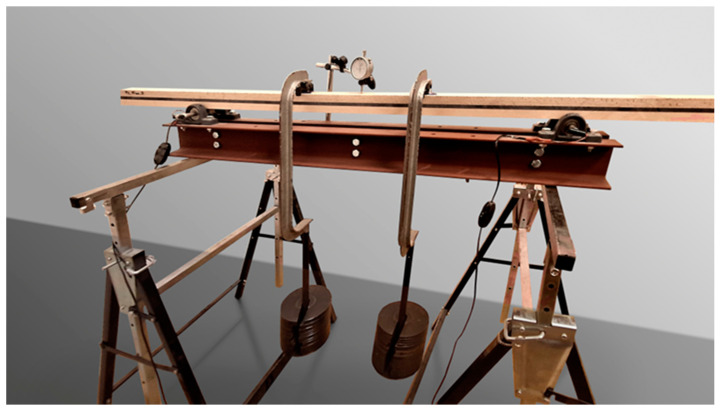
Laboratory setup for static four-point bending test.

The flexural stiffness of the composite beams was investigated using a static four-point bending test. The distance between supports was $L_{s}=1\;m$. Two point loads of 21 kg each were attached at 1/3 and 2/3 of the span length. For such a beam configuration, the flexural stiffness is calculated as follows:

$$EI=\frac{12}{1296}\frac{PL^{3}}{u}$$

where u is the midspan deflection. The midspan deflection was measured with a 2706102 dial gauge (HOREX, Germany), with a measurement range of 0.01–10 mm. The testing setup is presented in Figure 3. The results are summarized in Table 3. The measurement was performed on different specimens than those subjected to dynamic loading.

### 2.5. Analytical Estimates

#### 2.5.1. Discrete Mass Model and Application of the γ-Method

One of the most commonly used and effective mathematical methods for describing the mechanical responses of composite beams is the so-called γ-method [36,37]. It enables simple estimations of flexural stiffness—and, thus, deflections and stresses—for simple beams of two- or three-part cross-sections (I-sections, T-sections, or box sections) in which particular parts are joined with the use of deformable connectors. This method can be adjusted to describe deformable adhesive bondlines in a straightforward way. Although the original method was created for use on simply supported beams under a specific sinusoidally distributed load, it is also commonly used for other static schemes after proper modification of the span length (e.g., [38]). The composite cantilevers considered in this study may be regarded as a specific case of a two-part T-section, with the flange and web being the same (beech lamella). For cantilevers, the effective length should be twice the length of the beam.

The effective flexural stiffness of a composite beam, according to the γ-method, is estimated using Equation (2), with the parameters described in (3) and (4).

(2) $$EI_{eff}=\sum_{i=1}^{2}\left(E_{i}I_{i}+\gamma_{i}E_{i}A_{i}a_{i}^{2}\right),$$

where

(3) $$\gamma_{1}=\frac{1}{1+k_{1}},\;\;\gamma_{2}=1,\;\;k_{1}=\frac{\pi^{2}E_{1}A_{1}t}{b_{2}L^{2}G_{a}},$$

(4) $$a_{2}=\frac{\gamma_{1}E_{1}A_{1}\left(h_{1}+h_{2}\right)}{2\left(\gamma_{1}E_{1}A_{1}+\gamma_{2}E_{2}A_{2}\right)},\;\;a_{1}=\frac{h_{1}+h_{2}}{2}+t-a_{2}.$$

In the case of the considered cantilevers, we used $h_{1}=h_{2}=h$, $b_{2}=b$ and (5).

(5) $$A_{1}=A_{2}=bh,\;I_{1}=I_{2}=\frac{bh^{3}}{12}$$

When the flexural stiffness of the composite beam is known, it is easy to estimate the fundamental eigenfrequencies of a cantilever using the discrete mass model. The total mass of the beam is denoted by m, as defined by Equation (6).

(6) $$m=\int_{0}^{L}\mu dx=\int_{0}^{L}\left[2\rho_{w}bh+\rho_{a}bt\right]dx$$

**Figure 4:**
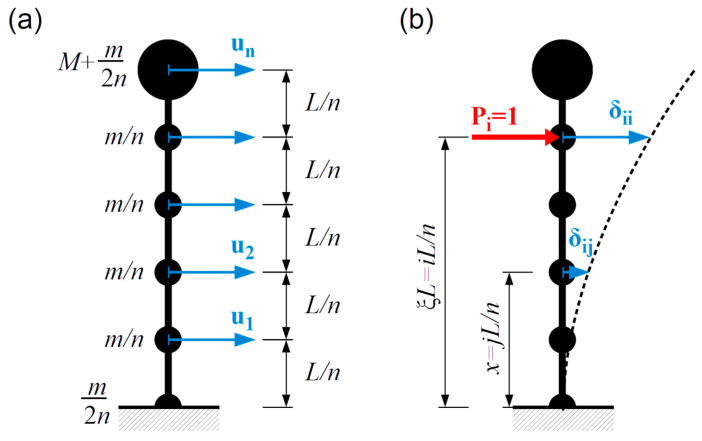
(**a**) Discrete mass model (**b**) Flexibility coefficients as displacements corresponding with a unit point load.

The total mass of the beam is uniformly divided into n lumped masses at equal distances $\frac{L}{n}$. The additional lumped mass attached to the free end of the cantilever is denoted by M (Figure 4a).

Standard structural dynamics procedures were applied to determine the eigenfrequencies of such a discrete model; details of the approach employing the flexibility coefficients are provided in [39]. The compliance matrix D was found to be a symmetric matrix, the entries $\delta_{ij}$ of which are displacements in the j-th point due to the application of a unit force in the i-th point (Figure 4b). These displacements are analytically found using (7).

(7) $$\delta \left(x\right)=\left\{\begin{array}{ll} \frac{PL^{3}}{EI}\left[\frac{\xi }{2}\;{\left(\frac{x}{L}\right)}^{2}-\frac{1}{6}{\left(\frac{x}{L}\right)}^{3}\right] & \Leftrightarrow x<\xi L\\ \frac{PL^{3}}{EI}\left[\frac{\xi^{2}}{2}\;\left(\frac{x}{L}\right)-\frac{\xi^{3}}{6}\right] & \Leftrightarrow x\geq \xi L \end{array}\right.$$

The mass matrix has a diagonal form and may be written as $M=diag\left(\frac{m}{n},\frac{m}{n},\ldots ,\frac{m}{2n}+M\right)$. The eigenfrequencies $\omega_{k}\;\left(k=1,\ldots ,n\right)$ are then found as solutions of the secular Equation (8).

(8) $$\det\left(DM-\frac{1}{\omega_{k}^{2}}1=0\right)$$

For example, for n=1, $\omega_{1}$ is presented in (9).

(9) $$\omega_{1}=\sqrt{\frac{3EI}{L^{3}\left(M+\frac{m}{2}\right)}}$$

The classical handbook on vibration by Weaver, Timoshenko, and Young [40] proposes a different contribution of the cantilever’s mass to the vibrating mass of the system:

(10) $$\omega_{1}=\sqrt{\frac{3EI}{L^{3}\left(M+\frac{33}{140}m\right)}}$$

For n=2, $\omega_{1}$ and $\omega_{2}$ are presented in (11) and (12), respectively.

(11) $$\omega_{1}=\sqrt{\frac{192EI}{L^{3}\left(32\;M+10\;m+\sqrt{1024\;M^{2}+584\;Mm+86{\;m}^{2}}\right)}}$$

(12) $$\omega_{2}=\sqrt{\frac{192EI}{L^{3}\left(32\;M+10\;m-\sqrt{1024{\;M}^{2}+584\;Mm+86\;m^{2}}\right)}}$$

For greater values of n, the analytical expressions become very complex, and for n>4, according to the Abel–Ruffini theorem, such expressions do not exist at all; however, the eigenfrequencies may be computed numerically.

#### 2.5.2. Dunkerley’s Estimate Based on a Linear Elastic Theory of Composite Beams

Another method of estimating the fundamental eigenfrequency is to use more accurate estimates of the flexural stiffness of a composite beam. The linear elastic theory of composite beams presented in [41,42] is employed. The maximal deflection of a cantilever loaded at its tip with a point force P according to the considered model is predicted by Equation (13), with the parameters outlined in (14).

(13) $$\delta =\frac{PL^{3}}{3\left(E_{1}I_{1}+E_{2}I_{2}\right)}\frac{\pi_{2}\lambda^{3}\left(e^{\lambda }+1\right)+12\pi_{1}\pi_{3}\left[\frac{\lambda }{2}\left(e^{\lambda }+1\right)-\left(e^{\lambda }-1\right)\right]}{\lambda^{5}\left(e^{\lambda }+1\right)}$$

where

(14) $$\pi_{1}=\frac{h}{2L},\;\;\pi_{2}=\frac{4G_{a}bL^{2}}{E_{w,L}bht},\;\;\pi_{3}=\frac{8G_{a}L^{3}b\left(h+t\right)}{t\left(E_{1}I_{1}+E_{2}I_{2}\right)},\;\;\lambda =\sqrt{\pi_{1}\pi_{3}+2\pi_{2}}$$

The formula for the deflection at an arbitrarily chosen point along the cantilever is extremely complex, due to the point load applied at the free end, so finding all components of the flexibility matrix D with this model is impractical. On the other hand, it is possible to find the lower bound estimate of the fundamental frequency using only the diagonal terms $\delta_{ij}\;(i=j)$. According to Dunkerley’s method (see, e.g., [43]), this estimate is determined using Equation (15).

(15) $$f_{D}=\frac{1}{2\pi }\sqrt{\frac{1}{\sum_{i=1}^{n}m_{i}\delta_{ii}}}\;$$

Then, the component $\delta_{ii}$ (displacements at the i-th point due to the unit point force applied at the i-th point) may be estimated as the deflections of a cantilever with a length equal to $L_{i}=i\frac{L}{n}$ according to Equation (13). Such an approximation is the exact solution in the case of a homogeneous, isotropic Bernoulli–Euler beam since the nonloaded part of the cantilever is not strained, and it deforms similarly to a rigid body and does not contribute to the overall flexural stiffness of the beam. This was not the case for the composite beam under consideration. While the global cross-sectional forces (for the beam as a whole) in the remaining part of the beam (from the point of application of the load to the free end) were indeed equal to 0, the local cross-sectional forces (for each lamella) were not—they constituted a self-equilibrated system of forces in which several axial forces in the lamella (of the same magnitude and in opposite directions) were equilibrated by bending moments acting on each of the lamella separately. All local cross-sectional forces were exactly equal to 0 only at the free end due to the traction-free surface boundary conditions. As a result, even if the composite cantilever was loaded in the middle point, the end part of the beam contributed to the global flexural stiffness. However, the analysis was simplified by neglecting this contribution and approximating the displacement due to the point force applied at the middle point by the displacement of a tip of a cantilever of an appropriately shorter length.

### 2.6. Numerical Modelling

The numerical analyses of the natural vibrations of cantilever composite beams were conducted using the finite element software Abaqus 2023 [44]. The problem was modeled as three-dimensional, and the bending vibration frequencies in both directions (parallel and perpendicular to the direction of the adhesive layer) were determined.

As the evaluated natural frequencies were dependent on the chosen element size, sensitivity tests were conducted. As a result, solid elements with a length of 2.5 mm were adopted, which led to a large problem size (over 250,000 elements).

In the first stage, the natural vibration problem of the reference wooden beam was modeled. A parametric analysis was conducted to study the relationship between the first bending eigenfrequency and the Young’s moduli of the orthotropic wood material (all Young’s moduli were scaled equally). Based on this analysis, Young’s moduli of the wood were selected to achieve a frequency in the radial direction consistent with the experimental results for the reference beam (see Table 2).

**Figure 5:**
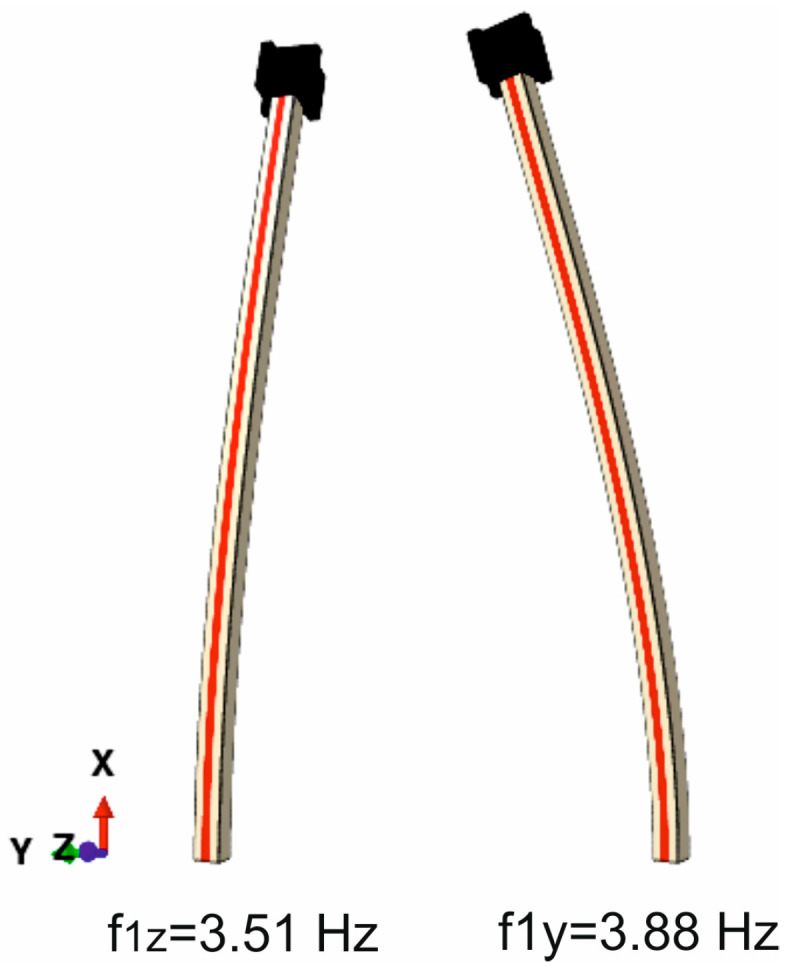
Fundamental eigenfrequencies and vibration modes in two perpendicular directions, Y and Z.

Using these data and assuming the linear–elastic behavior of the adhesive layer as per the material constants defined in Section 2.1.2, the dynamic characteristics of the composite cantilevers were determined (both without and with an attached mass). The sample mode shapes and natural vibration frequencies for a beam with a 15 mm thick adhesive layer and the attached mass are shown in Figure 5.

It may be observed that the lowest eigenfrequency is related to the bending mode in the plane parallel to the adhesive layer plane; this is most likely due to the lower flexural stiffness of the wooden lamella bent along the same plane compared to the lamella bent along the perpendicular plane. Composite beams are intended to be installed and loaded in such a way that the bending plane is perpendicular to the adhesive layer—for this reason only, these vibrations were investigated experimentally.

## 3. Results

### 3.1. Experimental Results

The results of the performed tests are presented in Table 4 for the sweep excitations and in Table 5 for the impact loads. It should be noted that while impact tests enable the identification of the natural frequency, sweep load tests enable the determination of resonance frequencies, which differ from the natural frequency of viscoelastic materials and should be slightly lower; it can be observed that, indeed, the resonant frequencies obtained from sweep tests (Table 4) are visibly smaller than the natural frequencies obtained for the same beams in impact tests. The tests are represented in the general format S/T/L/C, where S = B denotes a cantilever without additional mass, and S = B + M denotes a beam with an attached mass; T denotes the thickness of the adhesive layer in mm; L = S indicates sweep excitation; L = I denotes impact load; and C denotes the load case: C = HT—hitting the top of the beam with an impact hammer, C = HM—hitting the middle of the beam with an impact hammer, and C = M1 and C = M2—manual deflection of the beam during the first and second tests, respectively. C = *f*_1_ − *f*_2_*/L* stands for a sweep load in the range of frequency (*f*_1_; *f*_2_), and the load level *L* is a fraction of the reference acceleration—amplitude.

**Figure 6:**
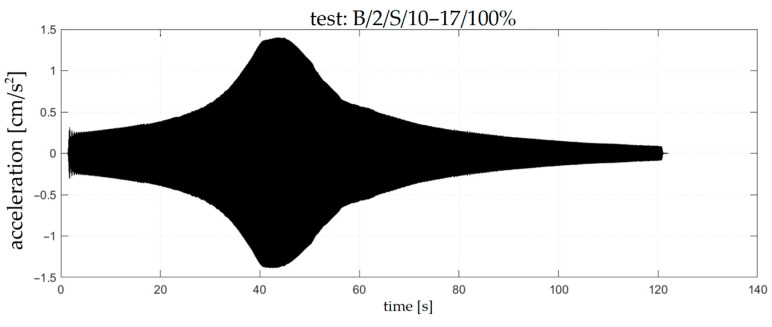
Accelerogram corresponding with B/2/S/10–17/100% test.

**Figure 7:**
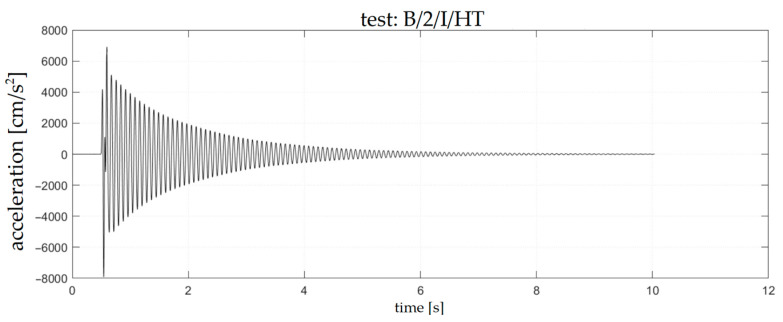
Accelerogram corresponding to B/2/I/HT test.

**Figure 8:**
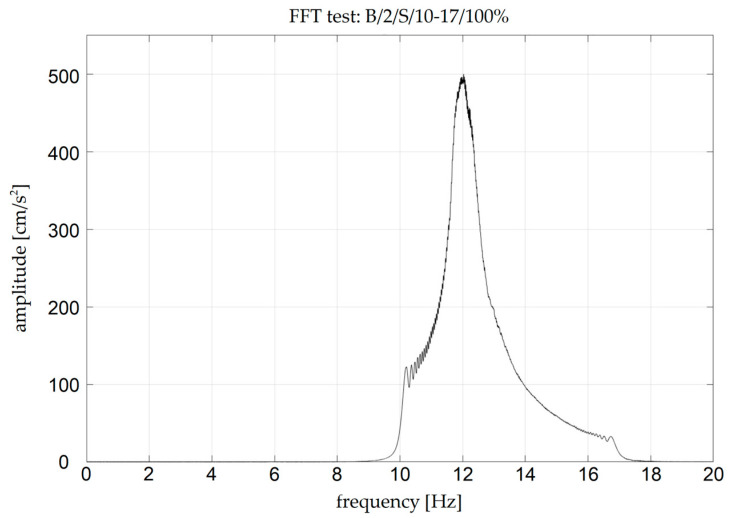
Fourier frequency spectrum corresponding to B/2/S/10–17/100% test.

**Figure 9:**
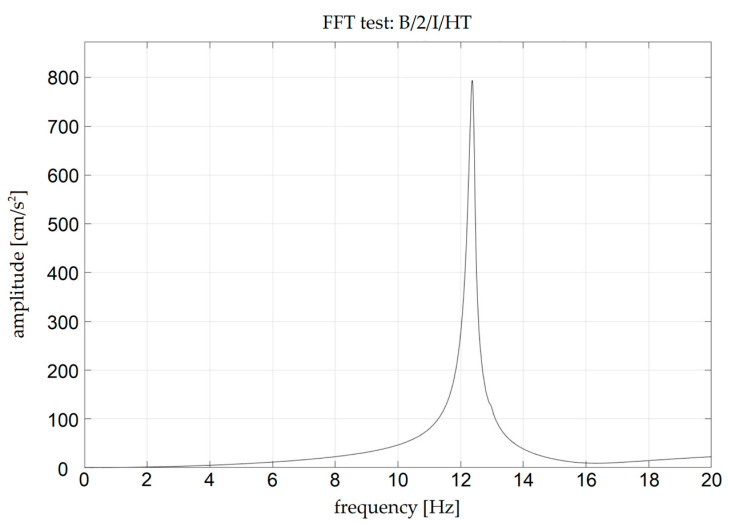
Fourier frequency spectrum corresponding to B/2/I/HT test.

Examples of accelerograms corresponding to the chosen sweep and impact tests are presented in Figure 6 and Figure 7, respectively. The frequency spectra corresponding to these accelerograms are presented in Figure 8 and Figure 9.

### 3.2. Comparison with Theoretical Predictions and Numerical Simulations

The experimentally measured fundamental frequency was the mean of all the results obtained from impact loading. Theoretical and numerical predictions of the fundamental natural frequencies $f_{0}$ of consecutive beams were created for undamped systems, and then the natural frequencies involving damping $f_{d}$ were adjusted according to the formula $f_{d}=f_{0}\sqrt{1-\gamma^{2}}$, in which the damping ratio γ was taken as the mean value of all damping ratios calculated according to the logarithmic decrement measured for each particular beam.

The natural frequencies of the analyzed beams can be estimated in several ways. The most simple approach is to employ the static flexural stiffness of the beam in the classical formulas for the eigenfrequencies of a cantilever [45]:

(16) $$f_{0}=\frac{3.5160}{2\pi L^{2}}\sqrt{\frac{EI}{\mu }}\;$$

The results for the cantilevers without a tip mass are summarized in Table 6.

**Figure 10:**
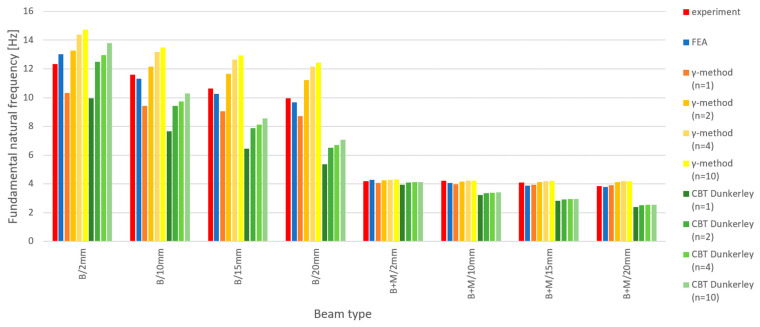
Measured and predicted fundamental frequencies of adhesively bonded beams.

The fundamental frequencies of adhesively bonded beams were also estimated using three methods: finite element analysis (FEA), solving the eigenproblem of structural dynamics by modeling the beam using the γ-method and estimating the lower bound using Dunkerley’s method, and approximating the beam’s flexibility with analytical solutions of linear composite beam theory (CBT). The accuracy of the latter two methods depends on the number of introduced degrees of freedom—cases corresponding to n=1, 2, 4 and 10 were analyzed. The obtained results are given in Table 7 and presented in Figure 10.

## 4. Discussion and Conclusions

In this study, the dynamic characteristics of adhesively bonded cantilevers were investigated experimentally and numerically. Eight types of specimens were analyzed: four adhesive layers with varying thicknesses were applied, and for each particular thickness, two beams—with and without an additional attached mass—were tested. Three types of excitations were considered: sweep load, impact load with a hammer, and the manual deflection of the beam. The natural frequencies were determined according to the frequency spectra of the recorded accelerograms, and the damping ratios were estimated with the use of logarithmic decrement. The numerical FEM model and two analytical approaches were used to predict fundamental eigenfrequencies based on the geometry of the system and the mechanical and physical properties of the used materials. According to the obtained results, we present the following conclusions:

- The sweep tests clearly indicated that the response of the adhesively bonded beam was nonlinear—the resonance frequencies were observed to drop as the amplitude of excitation increased. There are two main reasons for this observation. First, the deflection of the cantilevers vibrating due to sweep loads exceeded the magnitudes commonly accepted as small deformations—it was thus geometric nonlinearity that influenced the response of the beam in the case of large strains. Another cause is the nonlinear material characteristics of the polyurethane adhesive under consideration. While for small vibrations (as in the case of the response to an impact hammer), approximating the adhesive’s constitutive equations with Hooke’s relations does not introduce any significant error, in the case of large strains, it is necessary to take this physical nonlinearity into account.
- The FEA simulations matched the experimental results very well. For the very thin adhesive layer, the numerical predictions were somewhat overestimated, while in cases of greater adhesive thickness, the numerical simulations provided slightly underestimated natural frequencies. However, the differences did not exceed 5%.
- When the adhesive layer had a very small thickness, both analytical estimates were in good accordance with the FEA simulations and the experimental results. For greater thicknesses, the discrepancies were more significant, especially with regard to Dunkerley’s estimates using CBT.
- Analytical structural dynamics approaches utilizing the γ-method for determining the flexural stiffness of a composite cantilever tend to overestimate the fundamental frequency.
- The analytical method using the exact solutions of the linear composite beam theory for Dunkerley’s lower bound estimate provided strongly underestimated predictions for thick adhesive layers. In cases of thin bondlines, the approximation was good. Its advantage over the other presented approaches is that it does not require elaborations of an FEM model, nor does an eigenvalue problem need to be solved.
- The results of the performed research indicate that analytical estimates using the γ-method may provide reliable approximations if only a sufficient number of degrees of freedom are introduced in the discrete model. The composite beam theory was found to be successful only in the case of thin adhesive layers. The finite element linear perturbation (modal) analysis was found to be an accurate tool for predicting the natural frequencies of adhesively bonded beams even despite the relatively large deformations present in their dynamic response and despite the nonlinear mechanical characteristics of the adhesive.
- Further research will focus on analyzing other types of flexible polyurethane adhesives, the influence of deformation history (cyclic loads) on the mechanical properties of adhesives, and the global response of composite beams. The problem of nonlinear dynamic responses in cases of large deformations should also be the subject of an in-depth investigation.

## Figures and Tables

**Table 1:** Dimensions of cross-sections of composite beams.

Specimen Type	Height of Wooden Lamella $h\left[mm\right]$	Thickness of the Adhesive Layer $t\left[mm\right]$
B/2 mm	24	2
B/10 mm	20	10
B/15 mm	17.5	15
B/20 mm	15	20

**Table 2:** Mechanical properties of the beech wood.

Mechanical Properties of the Beech Wood	
Longitudinal Young’s modulus	$E_{w,L}=12.91\;GPa$
Radial Young’s modulus	$E_{w,R}=0.140\;E_{w,L}=1.79\;GPa$
Tangent Young’s modulus	$E_{w,T}=0.0767\;E_{w,L}=0.98\;GPa$
Kirchhoff’s modulus LR	$G_{w,LR}=0.120\;E_{w,L}=1.55\;GPa$
Kirchhoff’s modulus RT	$G_{w,RT}=0.0342\;E_{w,L}=0.44\;GPa$
Kirchhoff’s modulus LT	$G_{w,LT}=0.0788\;E_{w,L}=1.02\;GPa$
Poisson’s ratio LR	$\nu_{w,LR}=0.073$
Poisson’s ratio RT	$\nu_{w,RT}=0.36$
Poisson’s ratio LT	$\nu_{w,LT}=0.043$

**Table 3:** Results of the static four-point bending tests for span length L=1 m and load P=412 N.

Bondline Thickness t [mm]	Midspan Deflection *u* [mm]	Flexural Stiffness $EI\left[Nm^{2}\right]$
0 (solid beam)	1.37	5337.29
2	2.70	4687.23
10	2.48	3808.38
15	1.92	2948.42
20	1.56	2708.18

**Table 4:** Results of sweep tests.

Test Signature	Resonance Frequency f [Hz]	$\mathrm{Maximal}\;\mathrm{Amplitude}\;a_{max}$ [cm/s^2^]
B/2/S/10–17/100%	12.0297	500.1511
B/2/S/10–17/150%	11.7764	663.8601
B/2/S/10–17/200%	11.5434	770.4777
B + M/2/S/2–6/50%	3.8415	124.8115
B + M/2/S/2–6/100%	3.5255	131.9966
B + M/2/S/2–6/150%	3.2827	165.6727
B/10/S/10–17/100%	11.3132	341.9691
B/10/S/10–17/150%	11.2378	450.9628
B/10/S/10–17/200%	10.1819	517.9748
B + M/10/S/2–6/50%	4.1588	128.1414
B + M/10/S/2–6/100%	4.1849	142.6579
B + M/10/S/2–6/150%	3.4433	166.811
B/15/S/8–12/100%	10.367	536.321
B/15/S/8–12/150%	10.3171	669.9127
B/15/S/8–12/200%	10.5321	752.5345
B + M/15/S/2–6/50%	4.0571	137.4375
B + M/15/S/2–6/100%	3.5908	148.0918
B + M/15/S/2–6/150%	3.6499	165.1687
B/20/S/8–12/100%	9.6575	669.7734
B/20/S/8–12/150%	9.5655	890.1436
B/20/S/8–12/200%	9.6202	1032.5439
B + M/20/S/2–6/50%	3.7112	183.1002
B + M/20/S/2–6/100%	3.8283	202.531
B + M/20/S/2–6/150%	3.8143	217.6114

**Table 5:** Results of impact load tests.

Test Signature	Natural Frequency f [Hz]	$\mathrm{Maximal}\;\mathrm{Amplitude}\;a_{max}$ [cm/s^2^]	Logarithmic Decrement δ [-]	Damping Ratio γ [-]
B/2/I/HT	12.375	794.3143	0.0677	0.0108
B/2/I/HM	12.3671	878.3442	0.0562	0.009
B/2/I/M1	12.375	793.8866	0.0532	0.0085
B/2/I/M2	12.21	577.8685	0.0678	0.0108
B + M/2/I/HT	4.1697	105.5988	0.1168	0.0186
B + M/2/I/HM	4.1737	72.7273	0.0733	0.0117
B + M/2/I/M1	4.1583	173.6216	0.0539	0.0086
B + M/2/I/M2	4.2568	183.0667	0.0462	0.0074
B/10/I/HT	11.7321	501.2492	0.1087	0.0173
B/10/I/HM	11.7188	542.1618	0.1131	0.018
B/10/I/M1	11.4706	344.8527	0.1169	0.0186
B/10/I/M2	11.439	501.5453	0.1019	0.0162
B + M/10/I/HT	4.2339	138.3265	0.0969	0.0154
B + M/10/I/HM	4.2391	76.4188	0.4939	0.0784
B + M/10/I/M1	4.2366	164.1392	0.0538	0.0086
B + M/10/I/M2	4.2117	107.524	0.0658	0.0105
B/15/I/HT	10.6481	744.964	0.0847	0.0135
B/15/I/HM	10.6515	597.538	0.0878	0.014
B/15/I/M1	10.6452	637.2318	0.0829	0.0132
B/15/I/M2	10.6567	587.5158	0.0824	0.0131
B + M/15/I/HT	4.0843	124.1064	0.6892	0.109
B + M/15/I/HM	4.0873	92.318	0.5911	0.0937
B + M/15/I/M1	4.0829	146.2231	0.0626	0.01
B + M/15/I/M2	4.0795	158.7689	0.0639	0.0102
B/20/I/HT	9.971	697.5629	0.0742	0.0118
B/20/I/HM	9.96	818.4322	0.0773	0.0123
B/20/I/M1	9.9556	878.7887	0.0766	0.0122
B/20/I/M2	9.9438	856.5623	0.0653	0.0104
B + M/20/I/HT	3.8531	172.0414	0.7505	0.1186
B + M/20/I/HM	3.8544	87.5039	0.5866	0.093
B + M/20/I/M1	3.8527	191.3721	0.0531	0.0085
B + M/20/I/M2	3.8451	230.3046	0.0548	0.0087

**Table 6:** Measured and predicted fundamental frequencies (as a percentage of experimental results) of adhesively bonded beams, estimated using Equation (16) and the flexural stiffness from Table 3.

Fundamental Natural Frequency [Hz]		
Beam Type	Exp.	Equation (16)
B/2 mm	12.33	12.49 (101.3%)
B/10 mm	11.59	11.59 (100.0%)
B/15 mm	10.65	11.12 (104.5%)
B/20 mm	9.96	10.71 (107.5%)

**Table 7:** Measured and predicted fundamental frequencies (as a percentage of experimental results) of adhesively bonded beams.

Fundamental Natural Frequency [Hz]										
Beam Type	Exp.	FEA	γ-Method				Dunkerley Estimate for CBT			
			*n* = 1	*n* = 2	*n* = 4	*n* = 10	*n* = 1	*n* = 2	*n* = 4	*n* = 10
B/2 mm	12.33	13.02 (105.6%)	10.31 (83.6%)	13.29 (107.7%)	14.39 (116.7%)	14.73 (119.5%)	9.96 (80.8%)	12.50 (101.4%)	12.97 (105.2%)	13.81 (112.0%)
B/10 mm	11.59	11.33 (97.8%)	9.44 (81.4%)	12.16 (104.9%)	13.17 (113.6%)	13.49 (116.4%)	7.65 (66.0%)	9.42 (81.3%)	9.74 (84.0%)	10.30 (88.9%)
B/15 mm	10.65	10.26 (96.3%)	9.06 (85.1%)	11.67 (109.6%)	12.64 (118.7%)	12.94 (121.5%)	6.44 (60.5%)	7.87 (73.9%)	8.12 (76.3%)	8.57 (80.5%)
B/20 mm	9.96	9.69 (97.3%)	8.71 (87.5%)	11.23 (112.7%)	12.16 (122.1%)	12.45 (125.0%)	5.36 (53.8%)	6.51 (65.4%)	6.71 (67.4%)	7.07 (71.0%)
B+M/2 mm	4.19	4.27 (101.9%)	4.07 (97.1%)	4.24 (101.2%)	4.29 (102.4%)	4.30 (102.6%)	3.94 (94.0%)	4.08 (97.4%)	4.12 (98.3%)	4.14 (98.8%)
B+M/10 mm	4.23	4.05 (95.7%)	3.98 (94.1%)	4.17 (98.6%)	4.21 (99.5%)	4.23 (100.0%)	3.22 (76.1%)	3.35 (79.2%)	3.39 (80.1%)	3.40 (80.4%)
B+M/15 mm	4.09	3.88 (94.9%)	3.94 (96.3%)	4.14 (101.2%)	4.19 (102.4%)	4.21 (102.9%)	2.81 (68.7%)	2.92 (71.4%)	2.95 (72.1%)	2.96 (72.4%)
B+M/20 mm	3.86	3.77 (97.7%)	3.91 (101.3%)	4.12 (106.7%)	4.18 (108.3%)	4.19 (108.5%)	2.40 (62.2%)	2.51 (65.0%)	2.54 (65.8%)	2.55 (66.1%)

## Data Availability

The original contributions presented in this study are included in the article. Further inquiries can be directed to the corresponding author.
